# Control of *gdhR* Expression in *Neisseria gonorrhoeae* via Autoregulation and a Master Repressor (MtrR) of a Drug Efflux Pump Operon

**DOI:** 10.1128/mBio.00449-17

**Published:** 2017-04-11

**Authors:** Corinne E. Rouquette-Loughlin, Yaramah M. Zalucki, Vijaya L. Dhulipala, Jacqueline T. Balthazar, Raúl G. Doyle, Robert A. Nicholas, Afrin A. Begum, Erica L. Raterman, Ann E. Jerse, William M. Shafer

**Affiliations:** a Laboratories of Bacterial Pathogenesis, VA Medical Center, Decatur, Georgia, USA; b Department of Microbiology and Immunology, Emory University School of Medicine, Atlanta, Georgia, USA; c Emory Antibiotic Resistance Center, Emory University School of Medicine, Atlanta, Georgia, USA; d Departments of Pharmacology and Microbiology and Immunology, University of North Carolina, Chapel Hill, North Carolina, USA; e Department of Microbiology and Immunology, F. Edward Hébert School of Medicine, Uniformed Services University, Bethesda, Maryland, USA; Harvard Medical School

**Keywords:** gonococci, transcription, efflux pumps, physiology

## Abstract

The MtrCDE efflux pump of *Neisseria gonorrhoeae* contributes to gonococcal resistance to a number of antibiotics used previously or currently in treatment of gonorrhea, as well as to host-derived antimicrobials that participate in innate defense. Overexpression of the MtrCDE efflux pump increases gonococcal survival and fitness during experimental lower genital tract infection of female mice. Transcription of *mtrCDE* can be repressed by the DNA-binding protein MtrR, which also acts as a global regulator of genes involved in important metabolic, physiologic, or regulatory processes. Here, we investigated whether a gene downstream of *mtrCDE*, previously annotated *gdhR* in *Neisseria meningitidis*, is a target for regulation by MtrR. In meningococci, GdhR serves as a regulator of genes involved in glucose catabolism, amino acid transport, and biosynthesis, including *gdhA*, which encodes an l-glutamate dehydrogenase and is located next to *gdhR* but is transcriptionally divergent. We report here that in *N. gonorrhoeae*, expression of *gdhR* is subject to autoregulation by GdhR and direct repression by MtrR. Importantly, loss of GdhR significantly increased gonococcal fitness compared to a complemented mutant strain during experimental murine infection. Interestingly, loss of GdhR did not influence expression of *gdhA*, as reported for meningococci. This variance is most likely due to differences in promoter localization and utilization between gonococci and meningococci. We propose that transcriptional control of gonococcal genes through the action of MtrR and GdhR contributes to fitness of *N. gonorrhoeae* during infection.

## IMPORTANCE

The pathogenic *Neisseria* species are strict human pathogens that can cause a sexually transmitted infection (*N. gonorrhoeae*) or meningitis or fulminant septicemia (*N. meningitidis*). Although they share considerable genetic information, little attention has been directed to comparing transcriptional regulatory systems that modulate expression of their conserved genes. We hypothesized that transcriptional regulatory differences exist between these two pathogens, and we used the *gdh* locus as a model to test this idea. For this purpose, we studied two conserved genes (*gdhR* and *gdhA*) within the locus. Despite general conservation of the *gdh* locus in gonococci and meningococci, differences exist in noncoding sequences that correspond to promoter elements or potential sites for interacting with DNA-binding proteins, such as GdhR and MtrR. Our results indicate that implications drawn from studying regulation of conserved genes in one pathogen are not necessarily translatable to a genetically related pathogen.

## INTRODUCTION

*Neisseria gonorrhoeae* is the etiologic agent of the sexually transmitted infection (STI) termed gonorrhea, which is the second most prevalent bacterial STI in the United States and had a worldwide incidence of an estimated 78 million infections in 2012 (1). The capacity of gonococci to develop resistance to antibiotics is now of great concern with the recent emergence of strains resistant to current and past frontline antibiotics (2–5). With respect to the clinical efficacy of antibiotic treatment regimens, evidence has been presented that overproduction of the gonococcal MtrCDE efflux pump due to *cis*- or *trans*-acting mutations that elevate transcription of *mtrCDE* can contribute to clinically relevant levels of antibiotic resistance (6–10).

**FIG 1:**
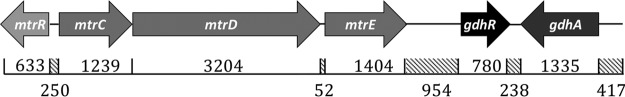
The organization of the gonococcal *mtr* and *gdh* loci in *N. gonorrhoeae* strain FA19, highlighting the position of genes relevant to this study. The length (shown in base pairs) and transcriptional orientation (direction of arrows) of relevant genes are shown. Numbers above the horizontal line refer to the coding regions of the gene, while the sizes of the intergenic regions (shaded boxes) are shown below. The distance between the two loci is 954 bp.

The *mtrR* gene, which encodes the master repressor (MtrR) of the *mtrCDE* efflux pump operon (8–10), is located immediately upstream of the *mtrCDE* operon (Fig. 1). The *mtrR* and *mtrCDE* genes are oriented away from each other and have overlapping promoters. Transcription of *mtrCDE* is repressed when MtrR is bound to the *mtrCDE* promoter, which overlaps the *mtrR* promoter (7, 8). Point mutations in the MtrR-binding site (8, 10), a single base pair deletion within a 13-bp inverted repeat sequence in the *mtrR* promoter (7), a point mutation that creates a new promoter (9), or missense mutations that cause radical amino acid replacements within the helix-turn-helix DNA-binding motif of MtrR can increase *mtrCDE* expression and antimicrobial resistance (8, 10). Such elevated expression of *mtrCDE* also increased gonococcal fitness *in vivo* when assessed by use of an experimental female murine lower genital tract infection model (11). In addition to regulating *mtrCDE*, MtrR serves as a global regulator of gonococcal genes (12) and directly or indirectly activates or represses at least 65 genes outside the *mtrCDE* locus. Included in these so-called “off-target” genes are those that are involved in the stress response (*rpoH*), amino acid synthesis (*glnA* and *glnE*), peptidoglycan biosynthesis (*ponA*), and regulation of gene expression (*farR*); the regulatory properties of MtrR have been summarized elsewhere (2, 13).

Our analysis of the whole-genome sequence of strain FA1090 (http://www.genome.ou.edu) revealed an open reading frame (termed NGO 1360) positioned 943 bp downstream of the *mtrCDE* operon that encodes a transcriptional regulator previously annotated GdhR in *Neisseria meningitidis* (Fig. 1). GdhR belongs to the bacterial GntR family of proteins, which serve as gene regulators and contain a highly conserved N-terminal DNA-binding domain and a variable C-terminal domain involved in effector binding and oligomerization (14). In *N. meningitidis*, which causes often deadly meningitis or fulminant septicemia (15), GdhR regulates the expression of a number of genes, some of which are involved in metabolism (16, 17). Meningococcal GdhR has been reported to activate *gdhA*, which encodes an NADP-specific l-glutamate dehydrogenase (16). Given the prominent role of MtrR in modulating gonococcal resistance to antimicrobials, its control of genes involved in metabolism, its influence on the fitness of gonococci in an experimental infection model, and its proximity to *gdhR*, we tested the capacity of MtrR to regulate expression of *gdhR* in *N. gonorrhoeae*, as well as the ability of GdhR to regulate genes in the *gdh* locus (*gdhR* and *gdhA*). Our results suggest that gonococcal and meningococcal GdhRs have distinct regulatory properties that are driven by differences in promoter utilization of regulated genes and emphasize the importance of bacterial species-specific studies for examining regulatory properties of a common DNA-binding protein.

## RESULTS

### The *gdh* locus in *N. gonorrhoeae*.

Similar to *N. meningitidis*, the *gdh* locus in *N. gonorrhoeae* FA19 is positioned 954 bp downstream from the *mtr* locus (Fig. 1) and contains two open reading frames, *gdhR*, which encodes a GntR-like DNA-binding protein, and *gdhA*, which encodes l-glutamate dehydrogenase. The gonococcal GdhR and GdhA proteins are 97% and 98% identical, respectively (data not shown) to the equivalent proteins described for meningococci (16). The end of *gdhA* is positioned 238 bp from the end of *gdhR* and is transcribed in the opposite direction; transcription of *gdhA* has been reported to be activated by GdhR in meningococci (16).

Bioinformatic analysis (http://www.ncbi.nlm.nih.gov) revealed that the 200 bp upstream of *gdhR* in five gonococcal strains (FA19, FA1090, MS11, FA6140, and F89) were identical, except for a C-to-T change in FA1090 29 bp upstream of the translation start codon but after the transcription start site (TSS) (see below). In these same gonococcal strains, 100% identity was noted for the 500-bp sequence upstream of *gdhA* (data not shown). When the same regions from *N. meningitidis* strain MC58 in the corresponding upstream regions of *gdhR* and *gdhA* were used in a BLAST search against whole-genome sequences, two other meningococcal isolates (LNP21362 and H44/76) were found to have identical sequences, while 10 others showed 99% identity (data not shown). Thus, our use of gonococcal strain FA19 for comparison to meningococcal strain MC58 is suitable for determining differences in regulation of the *gdh* locus in these pathogens. Although the DNA sequences of the *gdh* loci in gonococci and meningococci are very similar, important differences exist, especially in the location of promoters and potential *cis*-acting regulatory sequences (see Fig. 3 for the CE insertion in the meningococcal *gdhR* and see Fig. 6 for the *gdhA* promoters, respectively). We hypothesized that the differences in these sequences between gonococci and meningococci could impact GdhR-mediated regulation of gene expression in gonococci and influence gonococcal biology. Accordingly, we sought to identify a phenotype that is linked to GdhR production in gonococci and then to examine regulation of *gdhR* expression and the capacity of GdhR to control model genes.

### Loss of GdhR impacts *in vivo* fitness of gonococci independently of the *mtr* locus.

Given the close location of *gdhR* to the *mtr* locus (Fig. 1), we determined whether expression of GdhR influences transcription of *mtrCDE* and resistance of gonococci to antimicrobials recognized by the MtrCDE efflux pump (2, 6–12). For this purpose, we constructed a *gdhR* null mutant as well as a complemented strain. We found that the wild-type parent (FA19), the *gdhR*::*kan* mutant, and the complemented strain displayed identical levels of susceptibility to antimicrobials (erythromycin [Erm] MIC, 0.25 µg/ml; penicillin MIC, 0.015 µg/ml; Triton X-100 MIC, 100 µg/ml), and these MICs varied according to the levels of the MtrCDE efflux pump (6, 7, 11, 1,8). Moreover, results from quantitative reverse transcription-PCR (qRT-PCR) experiments indicated that expression of *mtrC* (the first gene in the *mtrCDE* operon [Fig. 1]) was not impacted by loss of GdhR (data not shown).

**FIG 2:**
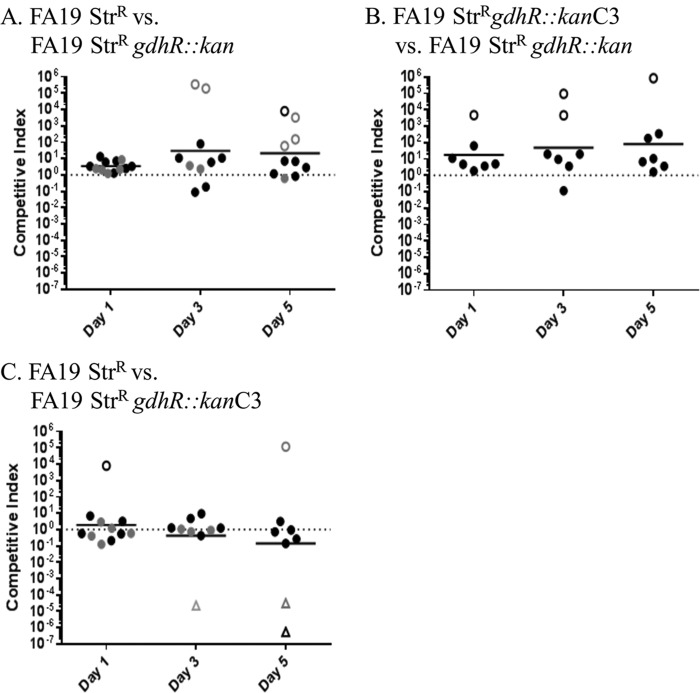
Absence of the GdhR protein confers a fitness advantage in *N. gonorrhoeae*. Competitive vaginal infections in female BALB/c mice with FA19Str^r^ and FA19Str^r^ *gdhR*::*kan* (A), FA19Str^r^ *gdhR*::*kan*C3 and FA19Str^r^ *gdhR*::*kan* (B), and FA19Str^r^ and FA19Str^r^ *gdhR*::*kan*C3 (C). Vaginal swab suspensions were quantitatively cultured on days 1, 3, and 5 post-bacterial inoculation, and the number of CFU of each strain was determined using selective agar as described in Materials and Methods. Each symbol corresponds to the CI from an individual mouse; open circles and open triangles correspond to mice from which only mutant CFU or wild-type CFU were recovered, respectively. Bars represent the geometric mean CI values. Combined data from two experiments are shown, with data points from each experiment indicated in black or grey. Open circles indicate that only the mutant strain was recovered from the vaginal swabs at the indicated time point. Open triangles indicate that only the wild-type strain was recovered from the vaginal swabs at the indicated time point. The differences in the median CI between the FA19Str^r^ versus FA19Str^r^ *gdhR*::kan C3 and the FA19Str^r^ *gdhR*::kan versus FA19Str^r^ *gdhR*::kan C3 competitions were statistically significant on day 1 (*P* = 0.02), day 3 (*P* = 0.07), and day 5 (*P* = 0.03) postinfection, based on the Kruskal-Wallis test with Dunn’s multiple comparisons test (GraphPad Prism). Comparisons of the FA19Str^r^ versus FA19Str^r^ *gdhR*::kan competition with FA19Str^r^ versus FA19Str^r^ *gdhR*::kan C3 showed that the results approached but did not reach a statistically significant difference; *P* values for days 3 and 5 in this comparison were 0.055 and 0.056, respectively.

We also assessed the fitness of the *gdhR* mutant relative to wild-type and complemented mutant bacteria during competitive infection of the lower genital tract of female BALB/c mice. Mice were inoculated vaginally with mixed bacterial suspensions containing similar numbers of the strains to be subjected to competition (total CFU per mouse, 10^6^), and the number of each strain of bacteria recovered from vaginal swab suspensions on days 1, 3, and 5 postinoculation was expressed as a competitive index (CI), as described in Materials and Methods. Ninety percent of the mice were colonized throughout the 5-day study period, with 10^4^ to 10^5^ CFU/vaginal swab suspension recovered from the majority of mice on day 1 postinoculation and >10^4^ CFU/ml on day 5 postinoculation (see Fig. S1 in the supplemental material). There was no difference in the relative fitness of the wild-type parental strain bacteria and the complemented *gdhR*::*kan* mutant strain (Fig. 2C). Interestingly, however, the *gdhR*::*kan* mutant strain was significantly more fit than the complemented *gdhR* mutant strain on days 1, 3, and 5 postinoculation (geometric mean CIs, 18, 49, and 81, respectively) (Fig. 2B) compared to the CIs for mice infected with the complemented mutant versus the wild-type strain (Fig. 2C). Competitive infections between the *gdhR*::*kan* mutant strain and the parental strain also showed elevated CIs (geometric mean CIs, 3.4, 30, and 21 on days 1, 3, and 5 postinoculation) (Fig. 2A), although the differences were not statistically significantly different from those for the complemented mutant strain versus the wild-type strain. Consistent with the *gdhR* mutant strain outcompeting the GdhR-expressing bacteria *in vivo*, only mutant CFU were recovered from some mice inoculated with wild-type or complemented mutant bacteria mixed with the *gdhR*::*kan* mutant bacteria on days 3 and 5 (Fig. 2A and B, open circles). These results indicated that GdhR production negatively influences the *in vivo* fitness of gonococci; importantly, loss of GdhR did not impact the growth rate of gonococci in GC broth (data not shown). This result suggested that GdhR may be a negative regulator of *in vivo* fitness; therefore, we sought to determine how *gdhR* is regulated in gonococci and if GdhR controls expression of model genes (*gdhR* and *gdhA*) previously studied in meningococci (16).

10.1128/mBio.00449-17.1 FIG S1  Colonization (in CFU per milliliter) determined from vaginal swab suspensions recovered from mice on days 1 through 5 postinoculation. Download FIG S1, TIF file, 10.6 MB . Copyright © 2017 Rouquette-Loughlin et al. 2017 Rouquette-Loughlin et al. This content is distributed under the terms of the Creative Commons Attribution 4.0 International license.

### MtrR is a direct repressor of *gdhR* expression.

**FIG 3:**
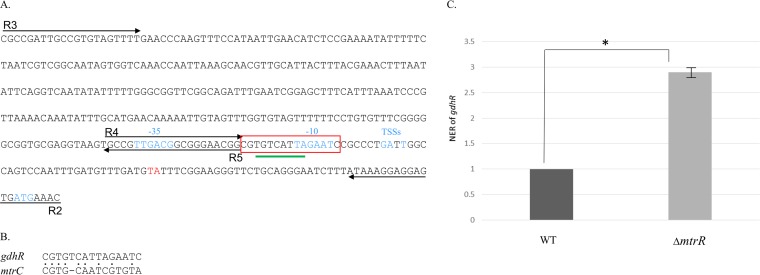
(A) Promoter sequence of the *gdhR* gene in strain FA19. The −10 and −35 promoter elements, the start of translation (ATG), and the TSSs are represented in blue. Primers are represented by arrows and the putative MtrR-binding site is framed in red. The insertion site of the CE in meningococci is represented in red (TA). The putative GdhR-binding site is underlined in green. (B) Alignment of the MtrR putative binding sites upstream of *gdhR* and *mtrC*. Dots between the two sequences indicate identical bases. (C) Quantitative RT-PCR results for *gdhR* in the wild type (WT) and a strain with *mtrR* deleted at the late-logarithmic phase of growth. Error bars represent standard deviations of the means of two independent experiments. Normalized expression ratios (NER) were calculated using 16S rRNA expression levels. *, *P* = 0.0004.

MtrR exerts transcriptional repression on the *mtrCDE* operon by binding to a promoter located upstream of *mtrC* (8, 19). Given the close proximity of *gdhR* to the *mtr* locus in both gonococci and meningococci, we asked if MtrR regulates *gdhR* expression in gonococci. Although *gdhR* was not previously assigned to be an MtrR-regulated gene in an earlier transcriptional profiling study that employed gonococcal RNA prepared from mid-logarithmic-phase cultures (12), we reexamined MtrR control of *gdhR* for two reasons. First, the presence of a putative MtrR-binding site upstream of the *gdhR* gene (Fig. 3A) suggested such control is possible. This MtrR-binding site (boxed in Fig. 3A) was 60% homologous to the MtrR-binding site on the *mtrC* promoter region (Fig. 3B). Second, the work of Mercante et al. (20) showed that a different transcriptional factor (MpeR) expressed in gonococci displays growth phase-dependent regulons.

**FIG 4:**
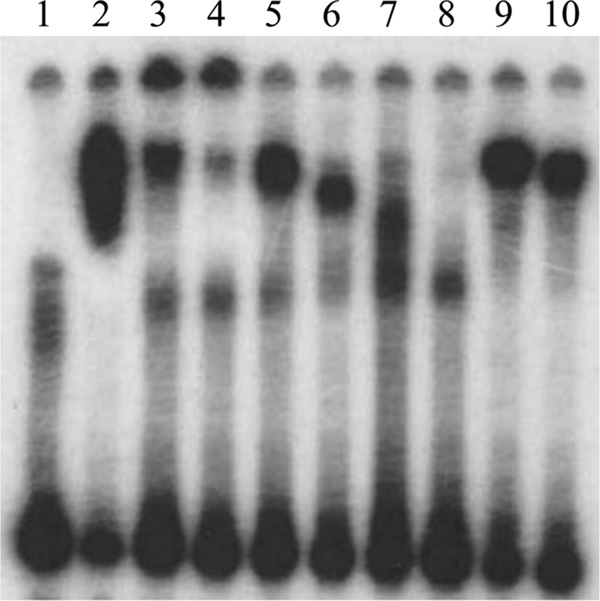
Competitive EMSAs. The MtrR-binding site located on fragment R4/R2 has the highest affinity for the MtrR protein. Lanes: 1, probe R3/R2* alone; 2, probe R3/R2* plus 2 μg of MtrR; 3, probe R3/R2* plus 2 μg of MtrR plus 50× unlabeled R3/R2; 4, probe R3/R2* plus 2 μg of MtrR plus 100× unlabeled R3/R2; 5, probe R3/R2* plus 2 μg of MtrR plus 50× unlabeled R3/R5; 6, probe R3/R2* plus 2 μg of MtrR plus 100× unlabeled R3/R5; 7, probe R3/R2* plus 2 μg of MtrR plus 50× unlabeled R4/R2; 8, probe R3/R2* plus 2 μg of MtrR plus 100× unlabeled R4/R2; 9, probe R3/R2* plus 2 μg of MtrR plus 50× *rnpB*; 10, probe R3/R2* plus 2 μg of MtrR plus 100× *rnpB*. An asterisk indicates a radioactive probe. The location of the different *gdhR* probes are shown in Fig. 3A.

Results from qRT-PCR experiments indicated that deletion of *mtrR* increases *gdhR* transcription, supporting the hypothesis that MtrR controls *gdhR* expression in gonococci by functioning as a repressor of this gene in the late-logarithmic phase of growth (Fig. 3C). Using primer extension (PE) analysis (see Fig. S2), we identified three *gdhR* TSSs, positioned 73, 72, and 70 nucleotides upstream of the start of translation of *gdhR*. These TSSs allowed us to identify a promoter element (5′-TAGAAT-3′ for the −10 hexamer and 5′-TTGACG-3′ for the −35 hexamer) 81 bp upstream of the ATG translational start codon (Fig. 3A). Importantly, the putative MtrR-binding site overlapped the −10 hexamer sequence of the predicted *gdhR* promoter. Based on this promoter mapping and the identification of a predicted MtrR-binding site within the putative *gdhR* promoter, we tested if MtrR bound in a specific manner upstream of the *gdhR* coding sequence, and we used an electrophoretic mobility shift assay (EMSA) for this purpose. We found that 2 µg of MBP-MtrR was sufficient to completely shift a ^32^P-labeled probe, termed R3/R2, that consisted of 393 bp of sequence upstream of the *gdhR* translational start (Fig. 4, lane 2). In order to better localize the MtrR-binding site(s) within this region, we performed a competitive EMSA with nonradioactive fragments of the R3/R2 probe used in the aforementioned EMSA. Binding competition assays showed that a smaller probe encompassing the *gdhR* promoter and its downstream region (probe R4/R2 [Fig. 3A]) competed with MtrR binding to the labeled R3/R2 probe (Fig. 4, lanes 3 and 4), while a fragment located upstream of the promoter (R3/R5 [Fig. 3A]) did not (Fig. 4, lanes 5 and 6). Accordingly, we propose that MtrR represses *gdhR* expression by binding within the promoter sequence that contains the predicted MtrR-binding site.

10.1128/mBio.00449-17.2 FIG S2  Results of primer extension analysis, showing the three identified *gdhR* TSSs. Download FIG S2, TIF file, 2.8 MB. Copyright © 2017 Rouquette-Loughlin et al. 2017 Rouquette-Loughlin et al. This content is distributed under the terms of the Creative Commons Attribution 4.0 International license.

### GdhR regulation of model genes in gonococci.

We selected two GdhR genes for study: *gdhR* and *gdhA*, which constitute the *gdh* locus (Fig. 1). We chose these 2 genes to test if *gdhR* is subject to autoregulation by its gene product and because *gdhA* has been reported to be a GdhR-activated gene in meningococci (16) and is positioned near *gdhR* in both pathogens.

**FIG 5:**
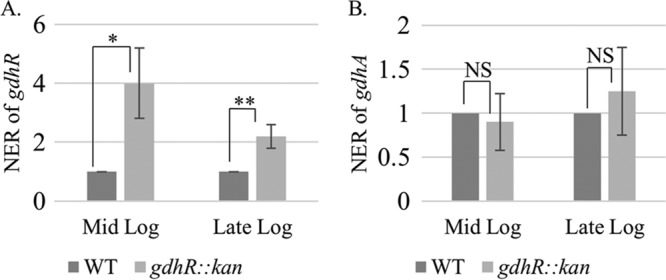
Quantitative RT-PCR results with *gdhR* (A) or *gdhA* (B) in wild-type (WT) and *gdhR-*negative strains at the mid- and late-logarithmic phases of growth. Error bars represent standard deviations from the means of three independent experiments. Normalized expression ratios (NER) were calculated using 16S rRNA expression. *, *P* = 0.011; **, *P* = 0.008; NS, not significant.

Pagliarulo et al. (16) suggested that the meningococcal *gdhR* transcript originates in a Correia element (CE) (21) located upstream of the *gdhR* gene. An examination of 23 publicly available gonococcal genome sequences revealed that this CE is absent in the *gdhR* promoter region (data not shown). However, a putative GntR-like binding site (5′-TGTCATTA-3′) was identified between the −10 and the −35 sites of the gonococcal *gdhR* promoter overlapping the predicted MtrR-binding site (Fig. 3A, underlined in green). In order to investigate autoregulation of *gdhR*, qRT-PCR analysis of total RNA prepared from mid- and late-log-phase cultures of strains FA19 and FA19 *gdhR*::*kan* was performed. The expression of *gdhR* was increased by 4-fold at mid-log phase and by a little more than 2-fold at late-log phase in the GdhR-negative mutant compared to the parental strain (Fig. 5A). We hypothesize that insertion of a CE upstream of *gdhR* in meningococci, but not in gonococci, results in the utilization of distinct *gdhR* promoters by these two related pathogens. Therefore, competing mechanisms of *gdhR* regulation by MtrR and GdhR itself may not occur in meningococci. In this respect, it is important to note that most meningococci encode an MtrR protein that contains loss-of-function mutations in *mtrR* (22, 23), while nearly 80% of gonococci encode a wild-type MtrR (reviewed in reference 2).

**FIG 6:**
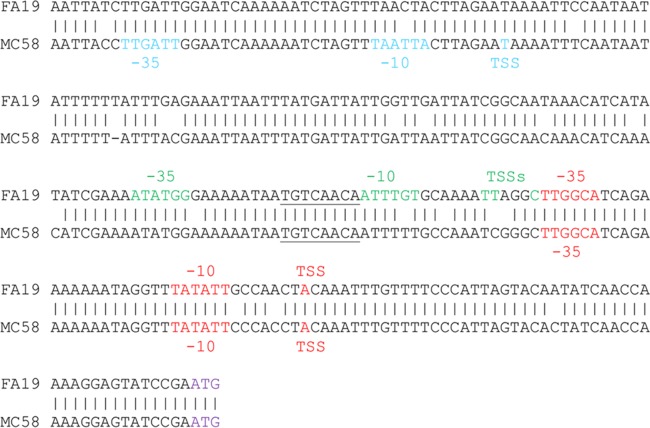
Alignment of *gdhA* promoters from gonococcal strain FA19 (top) and meningococcal strain MC58 (bottom). The TSSs determined by primer extension experiments for strain FA19 identified in this study and that of MC58 as reported by Pagliarulo et al. (16) are represented in blue, green, and red, with their respective putative promoter elements. The consensus binding sequence for the GntR protein is underlined. The *gdhA* translation start site is represented in purple.

**FIG 7:**
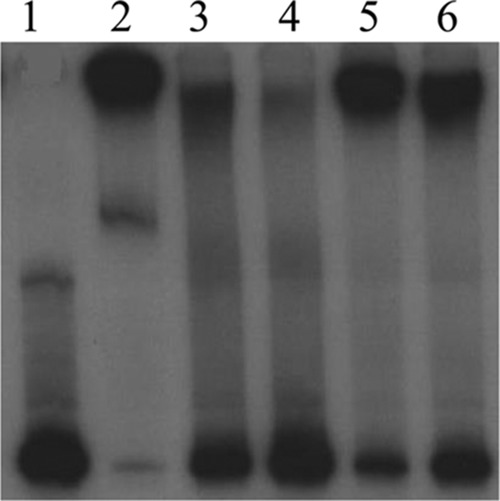
The GdhR protein binds to the *gdhA* promoter in a specific manner. Lanes: 1, probe PgdhA* alone; 2, probe PgdhA* plus 1 μg of GdhR; 3, probe PgdhA* plus 1 μg of GdhR plus 50× unlabeled PgdhA; 4, probe PgdhA* plus 1 μg of GdhR plus 100× unlabeled PgdhA; 5, probe PgdhA* plus 1 μg of GdhR plus 50× unlabeled *rnpB*; 6, probe PgdhA* plus 1 μg of GdhR plus 100× unlabeled *rnpB*. Asterisk refers to radioactive probe.

Previous work indicated that expression of *gdhA* in meningococci is directed by two promoters, only one of which is regulated by GdhR (16). The GdhR-activated promoter of *gdhA* in meningococci has a putative GdhR-binding site (5′-TGTCAACA-3′) upstream of the −35 hexamer, based on similarity to the known GntR-binding site (5′-TGTcaACA-3′; the lowercase letters refer to nucleotides that differ from consensus GntR-binding site) in other bacteria (14); this site is also located in the corresponding gonococcal sequence (underlined in Fig. 6). In order to investigate whether GdhR binds to this site in gonococci, as was shown previously in meningococci, we performed EMSA competition analysis using purified gonococcal His-tagged GdhR protein. The results showed that gonococcal GdhR binds specifically to a DNA fragment encompassing the GntR-binding site present upstream of *gdhA* in gonococci (Fig. 7).

In meningococci, the presence of another *gdhA* TSS was detected 207 bp upstream of the ATG translational start codon (represented in blue in Fig. 6). However, a GdhR-binding site was not identified within or near this distal promoter in meningococci. Interestingly, we did not detect a TSS 207 bp upstream of the ATG in gonococci. This could be due to the presence of a mutation which changes the −10 hexamer from 5′-TAATTA-3′ in meningococcal strain MC58 to 5′-TAACTA-3′ in gonococcal strain FA19. To determine if gonococci have an additional promoter(s) for *gdhA* transcription, we used PE analysis to identify transcription start sites. The results suggested the presence of two promoters (Fig. 6). We identified a TSS located 8 bp downstream from a −10 hexamer that constitutes the homolog of the above-mentioned meningococcal promoter (shown in red). We also identified three TSSs located upstream of a nonconsensus −10 hexamer (5′-ATTTGT-3′) that is spaced 17 nucleotides from a weak −35 hexamer (5′-ATATGG-3′) (represented in green in Fig. 6). Importantly, this putative promoter has the previously identified GdhR-binding site (underlined sequence in Fig. 6) between its −10 and −35 hexamer sequences. This second gonococcal promoter was not identified in meningococci. Based on the location of the two putative *gdhA* promoters in gonococci, GdhR could impact expression of *gdhA* from both promoters through interaction with the identified GdhR-binding site.

Taken together, our promoter mapping studies suggest that differences exist regarding *gdhA* transcription in gonococci and meningococci and that a GdhR-binding site influences *gdhA* transcription in gonococci. In order to assess promoter utilization in gonococci and any influence of GdhR on *gdhA* transcription, we performed qRT-PCR analysis on RNA prepared from strain FA19 and its isogenic *gdhR*::*kan* mutant at mid- and late-logarithmic phases of growth. Unlike its influence on *gdhR* expression, loss of GdhR did not impact *gdhA* expression in either mid-log- or late-log-phase cultures (Fig. 5B). One explanation for why we did not observe changes in *gdhA* expression is that GdhR binds upstream of the most proximal promoter (represented in red in Fig. 6) and inhibits the binding of the RNA polymerase to the second *gdhR* promoter (represented in green in Fig. 6), but it does not interfere with transcription from the proximal promoter, just as in meningococci. Consequently, when GdhR is present, the most proximal promoter (represented in red in Fig. 6) is the primary promoter used for transcription of *gdhA*. When GdhR is absent, the most distal promoter (green in Fig. 6) becomes the primary promoter for *gdhR* transcription.

## DISCUSSION

Our interest in *gdhR* was spurred by its close location to the *mtr* locus, which encodes the tripartite RND-type efflux pump MtrC-MtrD-MtrE and a transcriptional repressor (Fig. 1). We hypothesized that GdhR and MtrR might have cross-regulatory activities on the *mtr* and *gdh* loci, respectively. While we did not find evidence that GdhR regulates *mtrCDE* or antimicrobial resistance, we did find that its loss significantly increased fitness of gonococci during an experimental infection of the lower genital tract of female mice. This experimental model of infection has been used by us to show the importance of the MtrCDE pump for gonococcal survival *in vivo* and that gradients of fitness can be observed (11, 24), depending on the presence of distinct *cis*- or *trans*-acting mutations that influence *mtrCDE* expression (2).

Although the observed increase in fitness in the *gdhR*-negative mutant of strain FA19 was not as dramatic as previous findings (11) with an *mtrR*-negative mutant (5- to 10-fold versus ca. 100-fold), the impact on fitness was reproducible and significant. Therefore, we sought to define regulatory systems that influence *gdhR* expression in gonococci, and we compared our results with those obtained by others working on regulation of meningococcal genes controlled by GdhR. We discovered two *trans*-acting regulatory systems that were not previously observed in meningococci: (i) MtrR, which can directly repress expression of *gdhR* (Fig. 3), and (ii) GdhR, which can repress itself (Fig. 4). In the first instance, MtrR regulation of *gdhR* emphasizes the global regulatory properties of this DNA-binding protein in gonococci. It is noteworthy that this mechanism does not extend to most meningococcal isolates, since they harbor loss-of-function mutations in *mtrR* (22, 23). In the second instance, GdhR autoregulation of *gdhR* may be unique to gonococci, because the presence of a CE in this region in meningococci, but not gonococci, likely influences promoter utilization and GdhR binding.

GdhR has been previously studied in meningococci for its capacity to regulate genes involved in metabolism, but heretofore it has not been investigated for its regulatory properties in gonococci. Although GdhR has been reported to activate expression of *gdhA* and *gltT*, which encodes an l-glutamate transporter that appeared to be essential for full virulence in a rodent model of invasive meningococcal disease (25), its capacity to autoregulate its own gene or be controlled by *trans*-acting factors has not been elucidated in either pathogen. The work presented here illustrates that although two genetically related pathogens can encode the same transcription factor (e.g., GdhR) and have conserved target genes (e.g., *gdhA*), gene regulatory principles that have evolved for one pathogen may not necessarily apply to the related pathogen. Thus, although the GdhRs in meningococci and gonococci are identical, regulation of one target, *gdhA*, is distinct. While *gdhA* is a GdhR-activated gene in meningococci, based on quantitative analysis of levels of mRNA transcripts, our work failed to reveal differences in *gdhA* transcript levels in isogenic GdhR-positive and -negative gonococci. This does not mean that GdhR cannot activate *gdhA* in gonococci. We draw this conclusion because PE analysis suggested the presence of two promoters in gonococci that could direct transcription and be differentially impacted (activated or repressed) by GdhR, thereby giving the impression of lack of *gdhA* regulation. We propose that differences in the DNA sequence in the *gdh* locus in gonococci versus meningococci result in distinct promoter utilization and regulation.

Additional studies are needed to define the GdhR regulon in gonococci in order to understand the role of this DNA-binding protein in controlling genes important for metabolism and *in vivo* fitness of *N. gonorrhoeae*. In this respect, the increased fitness of the *gdhR* mutant observed on days 3 and 5 (Fig. 2) corresponds to the time inflammation is detected in the mouse model (26). With the protocol we use, proinflammatory cytokines and chemokines begin to increase on day 3 and peak on day 5, along with a peak polymorphonuclear leukocyte influx on day 5; expression of antimicrobial peptides also peaks on day 5 (A. E. Jerse et al., unpublished data). Thus, it is possible that *gdhR* may downregulate genes important in the invasion of innate defenses. Depression or induction of genes that are important in growth and metabolism could also contribute to the increased fitness observed with the *gdhR* mutant. With these possibilities in mind, which form the basis for future studies, our results emphasize that pathogen-specific regulatory actions of a common DNA-binding protein likely exist even between closely related bacteria (e.g., gonococci versus meningococci) and that differences in gene control, which could be influenced by *cis*-regulatory elements, may have consequences for the overall biology of members in same genus.

## MATERIALS AND METHODS

### Gonococcal strains, growth conditions, and determination of susceptibility to antimicrobial agents.

Strains used in this study are presented in Table 1. Gonococcal strains were grown overnight at 37°C under 5% (vol/vol) CO_2_ on GC agar containing defined supplements I and II (27). Determination of susceptibility of test strains to antibiotics was performed by the agar dilution method, and results were reported as the MIC (18). Antibiotics were purchased from Sigma Chemical Co. (St. Louis, MO). *Escherichia coli* strains were grown overnight at 37°C on LB agar.

**TABLE 1:** Strains of *Neisseria gonorrhoeae* employed in this study

Strain	Relevant genotype	Source(s)
FA19	Wild type	18
JF1	FA19 with *mtrR* deleted	27, 34
FA19Str^r^	FA19 with point mutation in *rpsL*	35
FA19Str^r^ *gdhR*::*kan*	FA19 with *rpsL aphA1* inserted in *gdhR*	This study
FA19Str^r^ *gdhR*::*kan*C3	FA19 with *rpsL aphA1* inserted in *gdhR* with wild-type copy of *gdhR* at *lctP-aspC* genomic locus	This study

### Construction of the *gdhR*-negative mutant and its complemented strain.

The plasmid construct used to insertionally inactivate the *gdhR* gene was created in pUC18us, which is pUC18 containing the 10-bp uptake sequence preceding the HindIII site in the polylinker. Overlap extension PCR was used to amplify the *gdhR* gene containing an internal XbaI site by using the upstream primer 5′GepR-new-Bam (5′-AGAGGATCCTAGAAACTGGTAAGGCCTCAGA-3′) and midstream reverse primer 3′GepR-mid-XbaI (5′-CTTCCTCAAACTTTTCTAGACAAAACCGAATCCGC-3′) to amplify the first half of the gene and the midstream forward primer 5′GepR-mid-XbaI (5′-GCGGATTCGGTTTTGTCTAGAAAAGTTTGAGGAAG-3′) and the downstream reverse primer 3′-gepR-EcoRI (5′-AGAGAATTCATACCTCCCAATCCTGCAC-3′) to amplify the second half of the gene. The two midstream primers are complementary to one another, and so in the second round of PCR, aliquots of each amplification product were used as the template with the forward upstream and reverse downstream primers to generate the *gdhR* gene containing an XbaI site in the middle of the gene. This modified *gdhR* construct was digested with EcoRI and BamHI and cloned into similarly digested pUC18us in which the existing XbaI site was destroyed by cutting with XbaI, filling in the 5′-overhangs with Klenow fragment, and religation. To create the *gdhR* inactivation construct, a blunt-ended kanamycin (Kan) resistance cassette derived from pLG338 (28) was ligated into the pUC18us-*gdhR* plasmid at the filled-in XbaI site in the middle of the *gdhR* gene. This construct was linearized by digestion with EcoRI and used to transform FA19Str^r^ (FA19 containing the *rpsL* allele from FA1090 that confers resistance to streptomycin [Str]), with transformants selected on GC agar plates containing 50 μg/ml Kan and verified by colony PCR and sequencing.

The pGCC3 vector (29) was used to complement FA19Str^r^ *gdhR*::*kan* because it allows the integration of a wild-type copy of *gdhR* under its own promoter at the transcriptionally silent intergenic region between *lctP* and *aspC*. pgntR3pac1 (5′-GATCTTAATTAAGCCGATTGCCGTGTAGTTTT-3′) and pme1gepR4 (5′-GATCGTTTAAACCCAGACCGTCTGAAC-3′) were used to amplify the *gdhR* gene. The resulting PCR product was cloned into the pGCC3 vector. The pGCC3*gdhR* construct was verified by sequencing and then transformed into FA19Str^r^ *gdhR*::*kan*. FA19Str^r^ *gdhR*::*kan*C3 transformants were selected on GC agar plates supplemented with 1 μg/ml of Erm and verified by colony PCR.

### Competitive murine infection.

Mixed bacterial inocula containing similar numbers of the two strains being tested were prepared by harvesting test strains from GC agar plates grown for 18 to 21 h and suspending the bacteria in 4 to 5 ml of 1× phosphate-buffered saline (PBS). The suspensions were passed through a 1.2-μm filter to remove bacterial aggregates and, using previously determined standard values for each strain relating readings of the optical density at 600 nm (OD_600_) to CFU counts, the bacterial suspensions were diluted to ~5 × 10^7^ CFU/ml before being mixed in a 1:1 ratio (actual ratios were determined by plating as described below). Female NCI BALB/c mice (6 to 8 weeks old; Charles River, Inc.) in the diestrus stage or anestrus were injected subcutaneously with 0.5 mg of Premarin (Pfizer) on days −2, 0, and +2. On day 0, the mice were inoculated vaginally with 20 μl of the mixed suspension (~1 × 10^6^ to 2 × 10^6^ CFU/mouse). Mice were also treated with Str, vancomycin, and trimethoprim as described elsewhere (30) to suppress the overgrowth of commensal flora that occurs under the influence of estrogen. The vaginas were gently swabbed with a PBS-moistened sterile swab on days +1, +3, and +5 postinfection, and the swab material was suspended in 1 ml of PBS. Serial dilutions were performed in GC broth with 0.05% saponin, and dilutions were plated on GC agar with 100 μg/ml of Str for determination of total CFU, GC agar with 100 μg/ml of Str and 50 μg/ml Kan for determination of FA19Str^r ^*gdhR*::*kan* CFU, or 1 μg/ml Erm for FA19Str^r^ *gdhR*::*kan*C3 CFU. Plates were incubated overnight at 37°C under 7% (vol/vol) CO_2_, and colonies were counted after 24 to 48 h. The number of CFU recovered on GC-Str plus Kan or GC-Str plus Em agar plates was subtracted from the number of CFU recovered on GC-Str agar to determine the number of wild-type/parent CFU recovered. The CI was calculated according to the following equation: [(CFU_Mutant_/CFU_Wt_)^Output^]/[(CFU_Mutant_/CFU_Wt_)^Input^]. A value of 20 CFU (limit of detection) was used to calculate the CI for cultures from which CFU from one of the two strains were not recovered. Competitive infections were repeated, and the data were combined to test reproducibility and increase the statistical power. The Kruskall-Wallis test with Dunn’s multiple-comparisons test (GraphPad Prism) was used to compare the differences in the CIs for mice inoculated with each mixture.

Animal experiments were conducted in the laboratory animal facility at USUHS, which is fully accredited by the Association for Assessment and Accreditation of Laboratory Animal Care, under a protocol approved by the USUHS Institutional Animal Care and Use Committee.

### Quantitative RT-PCR.

For qRT-PCR analysis of transcript levels, RNA was extracted from strains FA19, JF1 (FA19 Δ*mtrR*) (12), and FA19 *gdhR*::*kan* grown in GC broth with supplements to late-logarithmic phase by the TRIzol method as directed by the manufacturer (Thermo Fisher Scientific, Waltham, MA). Genomic DNA (gDNA) was removed by RNase-free DNase treatment and use of gDNA Wipeout (Qiagen, Germantown, MD). The resulting RNA was then reverse transcribed to cDNA using the QuantiTect reverse transcriptase kit (Qiagen). Quantitative real-time RT-PCR was performed using the generated cDNA, and results were normalized to 16S rRNA expression for each strain. Primers 16Smai_qRTF (5′-CCATCGGTATTCCTCCACATCTCT-3′) and 16Smai_qRTR (5′-CGTAGGGTGCGAGCGTTAATC-3′) were used for the 16S rRNA, while primers gdhR-qRT-R2 (5′-AACCGAATCCGCTTCAAATCGG-3′) and gepR_qRTF1 (5′-ATCAGGTATTGTCGGTATTGGAAG-3′) were used for the *gdhR* gene. Primers gdhA_qRTF (5′-TTCCATCAGGCGGTTGAAGAA-3′) and gdhA_qRTR (5′-TTTGTCGTCCTGCCAGGTTA-3′) were used for the *gdhA* gene. Both gdhR-qRT-R2 and gepR_qRTF1 anneal upstream of the kanamycin cassette insertion, allowing their use for qRT-PCR experiments that employ RNA extracted from the *gdhR*::*kan* mutant. Primers mtrC_qRT_F (5′-CGGATTTGGCGCGTTACAAA-3′) and mtrC_qRT-R (5′-TAATGCGCGAACGGTTCAGA-3′) were used for the *mtrC* gene. All qRT-PCRs were performed in experimental duplicates and biological duplicates or triplicates.

### Mapping transcriptional start sites by primer extension analysis.

Total RNA from strain FA19 was prepared at the late-logarithmic phase of growth in GC broth as described above, using the method of Baker and Yanofsky (31). Primer extension experiments were performed as described previously (7) with 6 μg of total RNA with primers PEgntR (5′-CCAGTTTCATCACTCCTCCT-3′) or PEgdhA (5′-TTTGAGGTTGGCAAACAGGG-3′). The AMV reverse transcriptase primer extension system from Promega (Madison, WI) was used as described by the manufacturer. The TSSs were determined via electrophoresis of the extension products on a 6% (wt/vol) DNA sequencing acrylamide gel adjacent to reference sequencing reaction mixtures.

### Purification of the GdhR protein.

Construction of pET15b*gdhR* was done by amplifying the *gdhR* open reading frame using the primers gdhR_F (5′-GATCGCCATATGAAACTGGTAAGGCCTCAG-3′) and gdhR_R (5′-GCGGATCCTCATACCTCCCAATCCTG-3′). The resulting PCR product along with the pET15b vector were digested with NdeI and BamHI, ligated overnight, and transformed into *E. coli* DH5α. The pET15b*gdhR* construct was confirmed by sequencing with vector-specific primers T7F (5′-TTAATACGACTCACTATAGG-3′) and T7R (5′-GCTAGTTATTGCTCAGCGG-3′).

For protein expression, pET15b*gdhR* was transformed into *E. coli* BL21(DE3) cells. Cultures (5 ml) of BL21(DE3)-pET15b*gdhR* cells were grown overnight at 30°C and added to 500 ml of LB broth the next morning. The culture was grown at 30°C until mid-log phase and then induced with 0.3 mM isopropyl-β-d-thiogalactopyranoside and grown overnight at 30°C. Cells were harvested and resuspended in 20 ml of 10 mM Tris (pH 7.5), 200 mM NaCl, and then EDTA-free protease inhibitor was added to the bacterial suspension. The cells were lysed by use of a French press cell as described elsewhere (32), membranes and unbroken cells were removed by centrifugation at 100,000 × *g*, and the supernatant was collected and filtered. GdhR-His was purified over a 2-ml nickel-nitrilotriacetic acid (Ni^+2^-NTA) column. After flowing the supernatant over the Ni^+2^-NTA column, the resin was washed successively with buffer containing 20 mM and 50 mM imidazole to remove contaminants and weakly bound proteins, and GdhR-His was eluted successively with buffer containing 100 and 200 mM imidazole. The fractions containing GdhR-His were concentrated and the imidazole-containing buffer was removed by dialysis into storage buffer (10 mM Tris-HCl [pH 7.5], 200 mM NaCl, and 1 mM EDTA). Dithiothreitol and glycerol were added to final concentrations of 1 mM and 10%, respectively. To verify the stability and purity of the GdhR and MtrR fusion proteins, we subjected 1 µg of purified proteins to sodium dodecyl sulfate-polyacrylamide gel electrophoresis (SDS-PAGE) using a 12% (wt/vol) polyacrylamide gel (33), and then stained the resolved proteins with Coomassie brilliant blue (CBB). Each protein preparation contained a single CBB-staining band; the respective proteins migrated in the SDS-PAGE gel with a molecular mass consistent with their fusion protein status (32.0 kDa for GdhR-His and 65 kDa for MtrR-MBP [data not shown]).

### EMSA.

DNA probes encompassing the *gdhR* or the *gdhA* promoter regions that were used in the EMSAs were amplified by PCR from FA19 genomic DNA using the upstream primer R3 (5′-CGCCGATTGCCGTGTAGTTTT-3′) or R4 (5′-TGCCGTTGACGGCGGGAACGG-3′) and the downstream primer R2 (5′-GTTTCATCACTCCTCCTTTAT-3′) or R5 (5′-CCGTTCCCGCCGTCAACGGCA-3′) for *gdhR* (relative to the direction of transcription) and P1958F (5′-GTTGTTGGCAATTTCAGCCCTT-3′) and P1358R (5′-CGTCATTCGGATACTCCTTTT-3′) for *gdhA*. When making radioactive probes, the indicated PCR products were labeled with [^32^P]dATP using T4 polynucleotide kinase (New England Biolabs). The labeled DNA fragments were incubated with 2 μg of MtrR-MBP, purified as described previously (8, 22), or with 1 μg of GdhR-His, in 30 μl of reaction buffer at room temperature. For the competition assays, the same nonlabeled probe or a nonlabeled PCR product along with rnpBF1 (5′-CGGGACGGGCAGACAGTCGC-3′) and rnpBR1 (5′-GGACAGGCGGTAAGCCGGGTTC-3′) primers were added to the reaction mixture. Samples were subjected to electrophoresis in a 6% native polyacrylamide gel at 4°C, followed by autoradiography.
