# Supplementary figures and images for: Control of gdhR Expression in Neisseria gonorrhoeae via Autoregulation and a Master Repressor (MtrR) of a Drug Efflux Pump Operon
Source: mBio. 2017 Apr 11;8(2):e00449-17. doi: 10.1128/mBio.00449-17 (PMC5388806; doi:10.1128/mBio.00449-17)

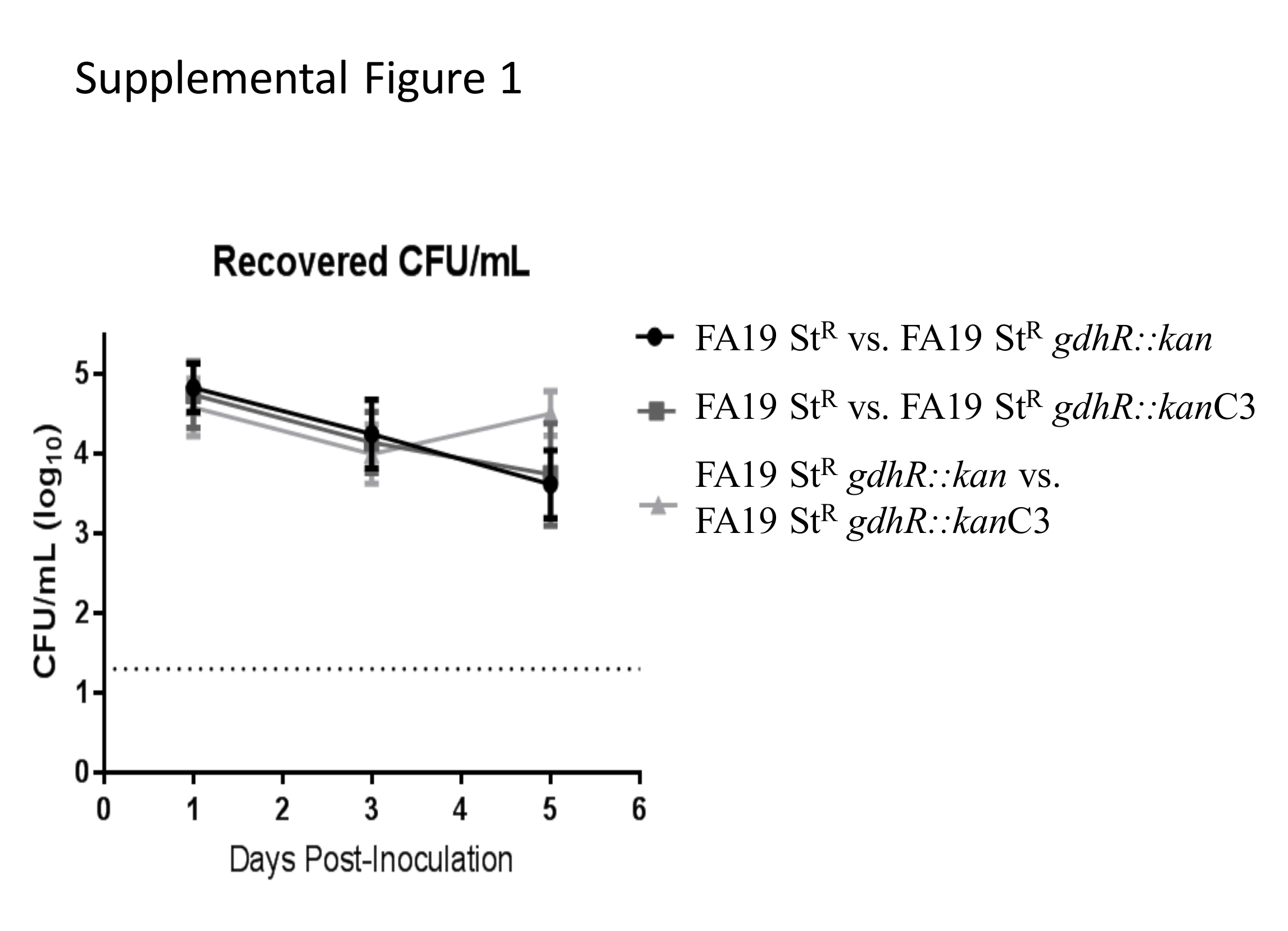

Supplement: FIG S1 [file mbo002173273sf1.tif]

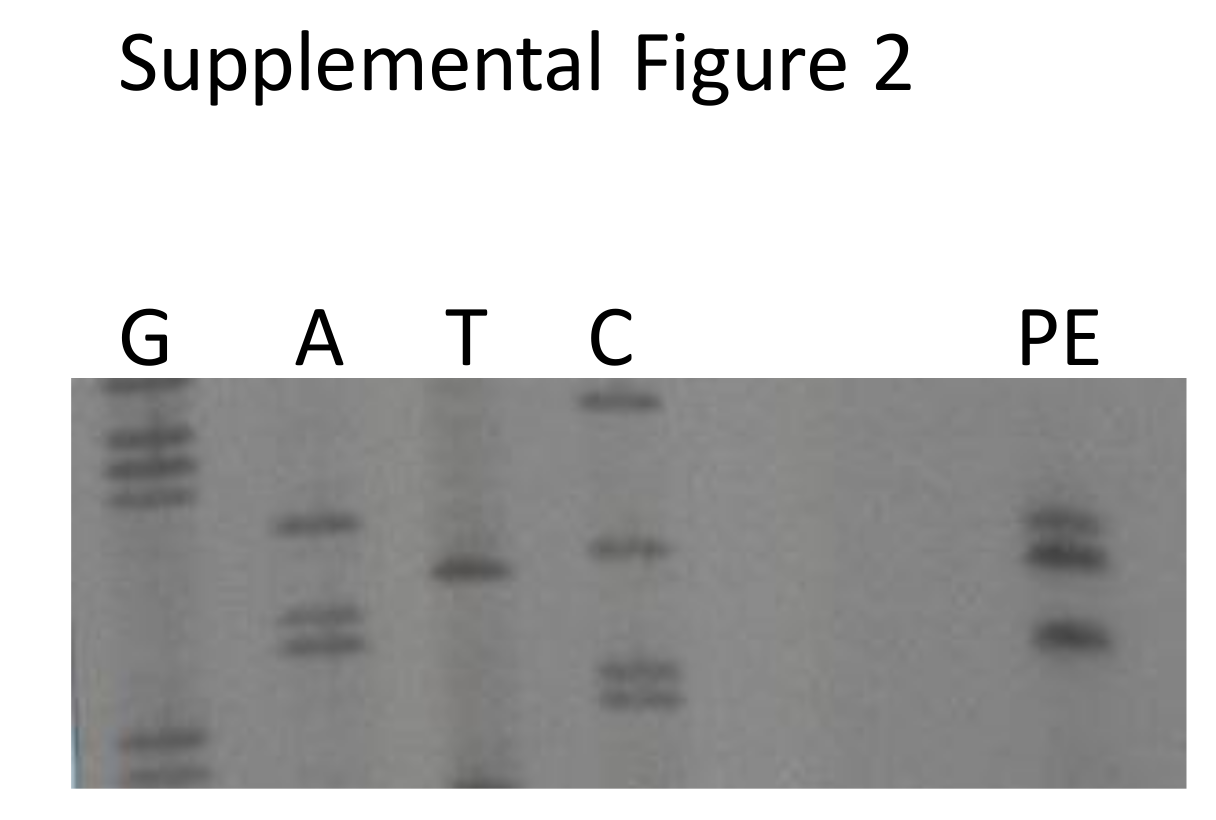

Supplement: FIG S2 [file mbo002173273sf2.tif]
